# New Data on Anti-Inflammatory and Wound Healing Potential of Transgenic *Senna obtusifolia* Hairy Roots: In Vitro Studies

**DOI:** 10.3390/ijms24065906

**Published:** 2023-03-21

**Authors:** Tomasz Kowalczyk, Przemysław Sitarek, Tomasz Śliwiński, Sophia Hatziantoniou, Nikolitsa Soulintzi, Rafal Pawliczak, Joanna Wieczfinska

**Affiliations:** 1 Department of Molecular Biotechnology and Genetics, University of Lodz, Banacha 12/16, 90-237 Lodz, Poland; tomasz.kowalczyk@biol.uni.lodz.pl; 2 Department of Biology and Pharmaceutical Botany, Medical University of Lodz, Muszynskiego 1, 90-151 Lodz, Poland; przemyslaw.sitarek@umed.lodz.pl; 3 Laboratory of Medical Genetics, Faculty of Biology and Environmental Protection, University of Lodz, Pomorska 141/143, 90-236 Lodz, Poland; tomasz.sliwinski@biol.uni.lodz.pl; 4 Laboratory of Pharmaceutical Technology, Department of Pharmacy, School of Health Sciences, University of Patras, 26504 Patras, Greece; sohatzi@upatras.gr (S.H.); nikol225@yahoo.gr (N.S.); 5 Department of Immunopathology, Medical University of Lodz, Zeligowskiego 7/9, Bldg 2, Rm 177, 90-752 Lodz, Poland; rafal.pawliczak@csk.umed.lodz.pl

**Keywords:** inflammation, *Senna obtusifolia* root extract, gene expression, airway cells

## Abstract

Asthma is an inflammatory disease whose etiology remains unclear. Its characteristics encompass a wide range of clinical symptoms, inflammatory processes, and reactions to standard therapies. Plants produce a range of constitutive products and secondary metabolites that may have therapeutic abilities. The aim of this study was to determine the effects of *Senna obtusifolia* transgenic hairy root extracts on virus-induced airway remodeling conditions. Three cell lines were incubated with extracts from transformed (SOA4) and transgenic (SOPSS2, with overexpression of the gene encoding squalene synthase 1) hairy roots of *Senna obtusifolia* in cell lines undergoing human rhinovirus-16 (HRV-16) infection. The effects of the extracts on the inflammatory process were determined based on the expression of inflammatory cytokines (IL-8, TNF-α, IL-1α and IFN-γ) and total thiol content. The transgenic *Senna obtusifolia* root extract reduced virus-induced expression of TNF, IL-8 and IL-1 in WI-38 and NHBE cells. The SOPSS2 extract reduced IL-1 expression only in lung epithelial cells. Both tested extracts significantly increased the concentration of thiol groups in epithelial lung cells. In addition, the SOPPS2 hairy root extract yielded a positive result in the scratch test. SOA4 and SOPPS2 *Senna obtusifolia* hairy root extracts demonstrated anti-inflammatory effects or wound healing activity. The SOPSS2 extract had stronger biological properties, which may result from a higher content of bioactive secondary metabolites.

## 1. Introduction

Plants are an inexhaustible reservoir of active compounds with a broad spectrum of action, and various phytotherapies have been used by most of the human population for thousands of years. Both extracts and individual compounds isolated from various parts of plants have found application in many disease entities [1]. The terpenoids, alkaloids, and related nitrogenous compounds and phenols produced by plants demonstrate various antimicrobial, anti-inflammatory, analgesic, antioxidant, anti-cancer, anti-diabetic, anti-obesity, and respiratory disease properties [2,3]. According to the World Health Organization (WHO), it is estimated that about 80 percent of the population in developing countries rely on traditionally-used medicinal plants for their primary health care [4]. Our previous studies showed that extracts from various plant species have biological effects in vitro and in vivo [5,6,7,8]. Interestingly, the combination of biotechnology in its broadest sense and biological studies have created an efficient platform for the large-scale extraction of valuable secondary metabolites with improved biological properties [9].

One key step in plant biotechnology is to obtain cell or tissue in vitro cultures that demonstrate increased production of biologically active compounds. A particularly important place in green biotechnology is occupied by hairy roots transformed by *Rhizobium rhizogenes*. By also applying various methods of manipulating metabolic pathways, it is possible to further enhance the production or accumulation of specific and medically important secondary metabolites [10].

To date, many species of in vitro plant cultures have shown desirable properties against a wide variety of human ailments. Notable among these is *Senna obtusifolia* L. of the *Fabaceae* family, also known as cassia, Chinese Senna, or java bean––an annual or perennial shrub that can grow up to two meters in height. The plant is native to tropical South America but is now invasive in many parts of Europe, America, Africa, Asia, Australia, and Oceania [11]. Our earlier studies have shown that transgenic hairy root extracts of *Senna obtusifolia* derived from unique in vitro cultures overexpressing the PgSS1 gene demonstrate stronger biological properties in many in vitro models than other roots [12].

Asthma is an inflammatory disease with an unclear etiology, though exposure to allergens or tobacco smoke as well as various genetic and environmental factors are known to be involved [13]. It is characterized by the presence of increased numbers of leukocytes, especially eosinophils, as well as lymphocytes in bronchial tissues. In both asthmatic and normal airway mucosa, the main cells are T lymphocytes; these are activated in response to antigen stimulation or during acute asthma exacerbations and produce large amounts of cytokines, such as Interleukin (IL-)3, IL-4, IL-5 or granulocyte-macrophage colony-stimulating factor (GM-CSF), which are significantly upregulated after antigen provocation, and their receptors have been identified locally on the surface of inflammatory cells [14]. Clinically, the symptoms of asthma include coughing, wheezing, shortness of breath, and chest discomfort [15]. The key mechanism in asthma is airway remodeling, which includes a range of pathophysiological elements, such as airway smooth muscle cell hypertrophy, extracellular matrix component (ECM) deposition, basement membrane growth, inflammatory cell infiltration, glandular hypertrophy, and hypertrophy and angiogenesis [16].

The effect of transgenic *Senna obtusifolia* hairy root extract on airway remodeling remains unknown, but our earlier studies indicate that curcumin, a polyphenolic compound extracted from *Curcuma longa* L., significantly reduced the expression of genes related to the airway remodeling process, which is dependent on NF-κB and, in part, on c-Myc and signal transducer and activator of transcription 3 (STAT3) [5]. In addition, apocynin, a natural compound isolated from *Alpine Himalayan*, affects the expression of selected genes involved in inflammatory and antioxidant responses in A549 cells (*IL-6, IL-8, tumor necrosis factor-α (TNF-α), PPAR-γ, thymic stromal lymphopoietin (TSLP), and CD59* [17]. Luo et al. showed that *Salvia miltiorrhiza* extract alleviates airway inflammation in ovalbumin (OVA)-sensitized allergic asthmatic mice [18]. In contrast, Guan et al. report that GRg1, the main active component of *Panax ginseng*, improves lung function, and protects against cigarette smoke-induced airway remodeling, in part by downregulating the transforming growth factor -β1 (TGF-β1)/Smad3 signaling pathway [19].

This study examines the effect of a unique *Senna obtusifolia* transgenic hairy root extract obtained by agrotransformation using *Rhizobium rhizogenes* on virus-induced airway remodeling by altering the levels of gene expression for inflammatory cytokines (IL-8, TNF-α, IL-1α and IFN-γ), measured using qPCR and levels of these proteins by enzyme-linked immunoabsorbent assay (ELISA). The results may contribute to the development of new therapeutic strategies for treating respiratory diseases in the future. The study also analyzes the effect of the extracts on gingival fibroblasts, comparing the findings with those obtained on different types of cells. Moreover, it evaluates the wound healing properties of the extracts by migration of NIH3T3 mouse fibroblasts using the wound healing scratch assay.

## 2. Results

### 2.1. Phytochemical Analysis

Consistent with our previous analyses, anthraquinones and pentacyclic triterpene, such as betulinic acid, were found in the studied extracts [12,20]. However, a higher content of betulinic acid was detected in the extract prepared from the transgenic hairy roots of *S. obtusifolia*.

### 2.2. Cell Viability after Treatment with SOA4 and SOPSS2 Root Extracts from Senna Obtusifolia

**Figure 1:**
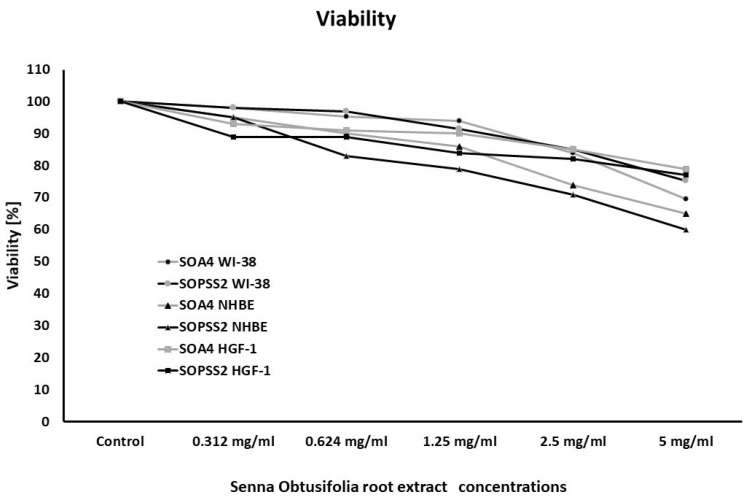
Viability of WI-38, NHBE and HGF-1 cells after exposure to extracts from transformed (SOA4) and transgenic (SOPSS2) *Senna obtusifolia* hairy roots. Cell viability was assessed after 24 h. The values represent means.

*Senna obtusifolia* hairy root extracts showed no toxicity towards any of the cell lines used. Airway epithelial cells (NHBE) appeared to be more sensitive to the effects of the extracts than airway fibroblasts, while gingival fibroblasts were the least sensitive. No statistically significant differences were observed between cell viability after incubation with transformed (SOA4) and transgenic (SOPSS2) extracts of *Senna obtusifolia* (Figure 1).

### 2.3. Immunomodulatory Effects

**Figure 2:**
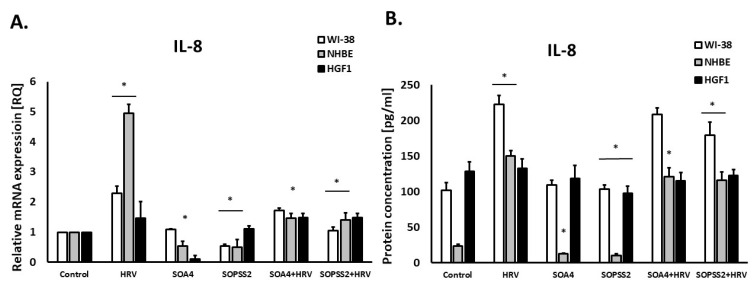
The effect of the extracts from transformed (SOA4) and transgenic (SOPSS2) *Senna obtusifolia* hairy roots and viral infection (HRV-16) on the expression of *IL-8* on mRNA and protein levels in human lung fibroblasts, epithelial cells, and gingival fibroblasts. (**A**) presents mRNA expression of IL-8; (**B**) presents protein concentration in airway fibroblasts (WI-38), epithelial cells (NHBE) and gingival fibroblasts (HGF1).Data presented as mean ± SEM, * *p* < 0.05.

The extracts from the transformed (SOA4) and transgenic (SOPSS2) *Senna obtusifolia* hairy roots showed a strong inhibitory effect on interleukin 8 expression in epithelial cells (*p* < 0.05). The extracts also hindered HRV-induced IL-8 release (*p* < 0.05, Figure 2A,B). In airway fibroblast cells, the reduced IL-8 expression was only noted after incubation with SOPSS2 extract.

Interestingly, IL-8 expression was also reduced in HGF1 gingival fibroblasts (*p* < 0.05), but only under the influence of SOPSS2 extract (*p* < 0.05), with no impact observed for SOA4.

**Figure 3:**
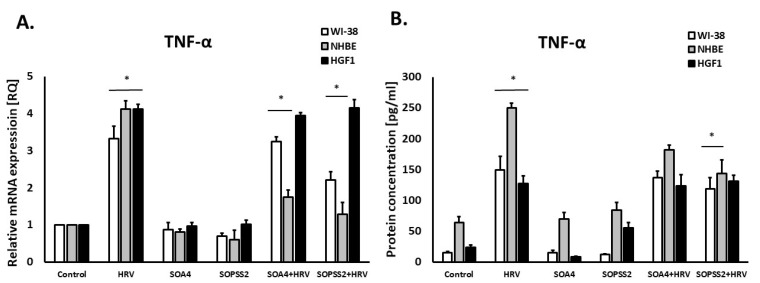
TNF-α expression in WI-38, NHBE and HGF-1 cells after treatment with SOPSS2 and SOA4 extracts from *Senna obtusifolia* under the conditions of HRV-16 infection. (**A**) mRNA and (**B**) protein level of TNF-α expression. Extracts from transformed (SOA4) and transgenic (SOPSS2) *Senna obtusifolia* hairy roots. Data presented as mean ± SEM, * *p* < 0.05.

It was also found that SOPSS2 (*p* < 0.05), but not SOA4, significantly reduced rhinovirus-induced TNF-α expression in both airway fibroblasts and epithelial cells, with no effect in gingival fibroblasts (Figure 3A,B).

**Figure 4:**
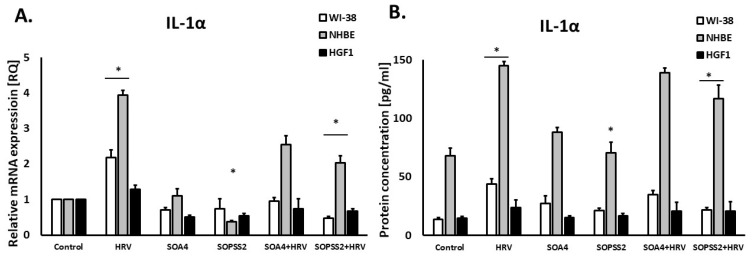
The effect of the extracts from transformed (SOA4) and transgenic (SOPSS2) *Senna obtusifolia* hairy roots and viral infection (HRV-16) on the expression of *IL-1α* on mRNA (**A**) and protein (**B**) levels in human cells. Data presented as mean ± SEM, * *p* < 0.05.

**Figure 5:**
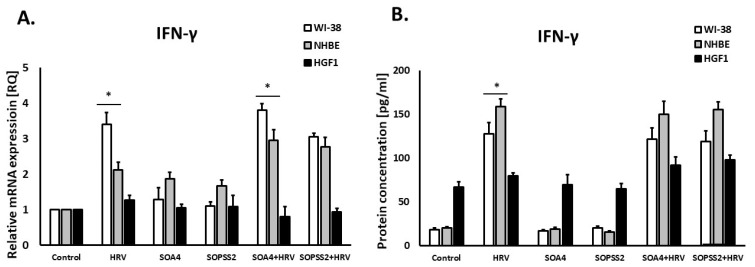
The lack of effects of SOA4 and SOPSS2 extracts from *Senna obtusifolia* roots on IFN-γ expression on mRNA (**A**) and protein (**B**) levels. Extracts from transformed (SOA4) and transgenic (SOPSS2) *Senna obtusifolia* hairy roots. Data presented as mean ± SEM, * *p* < 0.05.

SOPSS2 reduced IL-1α expression in epithelial cells (*p* < 0.05), but SOA4 did not. In addition, SOPSS2 inhibited rhinovirus-induced expression of IL-1α in both airway fibroblasts and epithelial cells (Figure 4A,B). None of the *Senna obtusifolia* extracts had any effect on IL-1α expression in gingival fibroblasts. No significant results were obtained in case of interferon-γ expression in either cell line (Figure 5A,B).

### 2.4. Effect of SOPSS2 Hairy Root Extract of Senna obtusifolia on the Migration of Fibroblast Cells

**Figure 6:**
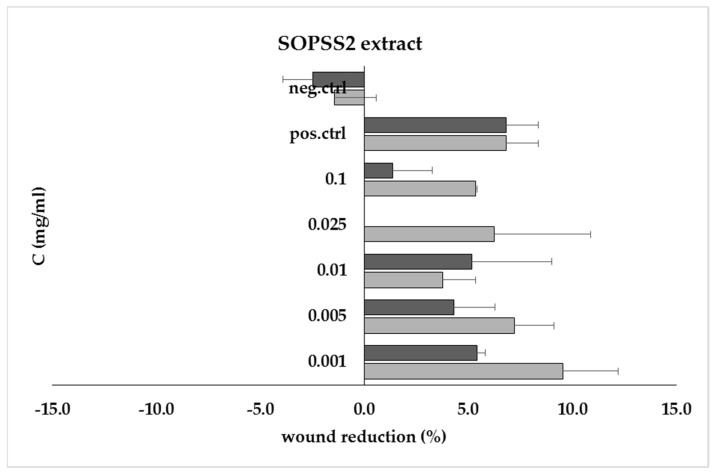
Wound recovery (% scratch reduction) at 24 h (■) and 48 h (■) for SOPSS2 root extract at concentrations ranging from 0.001 mg/mL to 0.1 mg/mL, compared to positive (pos.ctrl) and negative (neg.ctrl) controls.

As cell migration plays a key role in wound healing, a scratch test was performed to observe the healing process of the test sample. The SOPPS2 extract was used in this study because of its stronger biological properties revealed in previous studies. Our results showed that the 0.001 mg/mL extract demonstrated positive migration/proliferation properties after 24 h that were comparable to positive control (Figure 6).

### 2.5. The Effect of SOA4 and SOPSS2 of Senna obtusifolia Root Extracts on Thiol Groups

**Figure 7:**
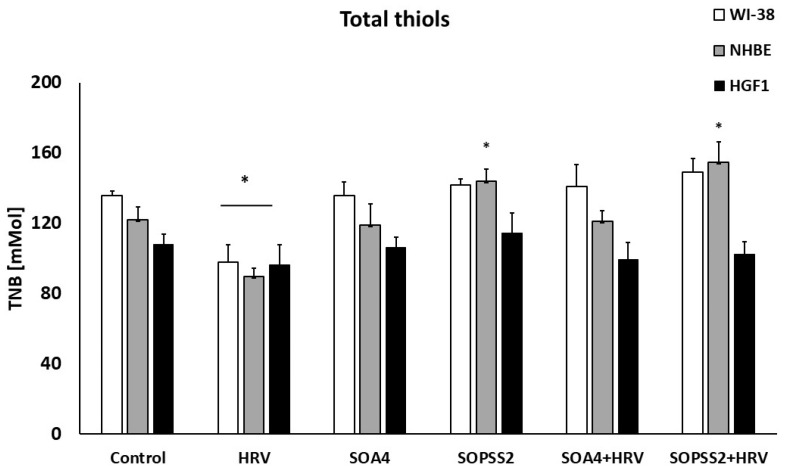
SOPSS2 induced the concentration of thiols in HRV infection conditions. Extracts from transformed (SOA4) and transgenic (SOPSS2) *Senna obtusifolia* hairy roots are given. Data presented as mean ± SEM, * *p* < 0.05.

To determine the effect of *Senna obtusifolia* hairy root extracts on oxidative balance, the concentration of thiol groups in cell cultures was determined. The extract from the transgenic culture induced statistically significant changes in the concentration of thiol groups, with this effect only visible in epithelial cells. No such changes were noted for the transformed root extract. In addition, the transgenic extract increased the concentration of thiol groups than had been reduced by HRV (Figure 7).

## 3. Discussion

Although HRV infection has mainly been associated with asthma exacerbations, rhinoviruses can cause asthma-like alterations in the lungs prior to allergen contact [21,22,23,24]. This is supported by numerous clinical investigations that show that repeated wheezing episodes can frequently result from multiple infections with HRVs in preschool-aged non-asthmatic children [24,25,26].

In asthma patients, fibroblasts are found in greater numbers in the submucosal region of the airway. These fibroblasts are actively involved in subepithelial fibrosis, exhibiting an invasive proliferative and secretory character that increases ECM deposition. Therefore, they were suitable candidates for determining the effect of treatment in the present study. Key profibrogenic mediators TGF-β and TNF-α are generated in large quantities during airway remodeling [27]. Studies conducted in vitro have shown that TGF-β activation promotes fibroblast proliferation and, when combined with TNF-α, facilitates the conversion of fibroblasts to fibrogenic, highly-secretory myofibroblasts. It has also been demonstrated that TNF-α exerts direct or indirect chemokine secretory activity that triggers the recruitment of inflammatory cells into the airways [28,29].

In this study, for the first time, rhinovirus was used to trigger changes of airway remodeling to examine the effects of both *Senna obtusifolia* transgenic hairy root extract with overexpression of the squalene synthase 1 (a gene that is associated with the production of terpenoids) and transformed hairy root extract on cytokine expression in human cells in vitro model. *Senna obtusifolia (Fabaceae*) is a well-known traditional Chinese medicinal plant with multidirectional effects. Numerous studies show an anti-inflammatory effect by altering cytokine levels in cells [30].

In the present study, the studied extract from transgenic hairy roots of *Senna obtusifolia* caused a reduction in TNF-α expression in both epithelial cells and fibroblasts. In addition, the extract caused a reduction in rhinovirus-induced TNF-α expression. It has been proven that patients with severe asthma express more TNF-α [31], and a unique TNF-α phenotype has recently been reported in children with moderate-to-severe asthma [32,33]. Therefore, the ability of the tested transformed and transgenic *S. obtusifolia* extracts to reduce TNF-α expression and concentration appears to be a promising result worthy of further analysis, particularly since TNF-α is a strong proinflammatory cytokine that controls airway inflammation. A growing body of research has shown that TNF-α may stimulate the release of inflammatory proteins by activating a number of signaling pathways, including STAT3 [34,35].

The literature shows that secondary metabolites contained in *S. obtusifolia* extracts can reduce inflammatory cytokine levels in various in vitro models [36]. Our studies revealed that transformed and transgenic hairy root extracts of *S. obtusifolia* decreased IL-8 expression in both airway cell lines but also reduced HRV-induced expression in epithelial cells. Elevated IL-8 levels in the airway secretions of both asthmatic and chronic obstructive pulmonary disease (COPD) patients may be indicators of a continuing inflammatory process that is more apparent in COPD patients. Kim et al. showed that an aqueous extract of *C. obtusifolia* L. inhibited interleukin (IL)-6 and reduced activation of the nuclear transcription factor-kB p65 in colon tissues [37]. We suggest that the biologically active compounds contained in the tested extracts, among which betulinic acid (BA) was present in higher amounts in the transgenic roots compared to the transformed roots without the genetic construct, may be responsible for extract activity, but other compounds such as anthraquinones, for example, may give an additional synergistic effect. In vitro and in vivo studies revealed that betulinic acid has beneficial biological properties [38,39,40,41]. The application of BA reduced the release of lipopolysaccharide (LPS)-triggered TNF-α, raised the amount of IL-10 in serum, and protected from a fatal dosage of LPS. Additionally, in vitro tests revealed that BA reduced the synthesis of TNF-α and NO in LPS-activated macrophages and increased the production of IL-10 [42]. Previous studies have also noted that BA inhibits the production of IL-8 as well as various other critical pro-inflammatory cytokines (TNF, IL-1β, IL-6) [42,43,44,45]. Most of these may be connected to the suppression of the NF-κB transcription factor, which plays a wide range of roles in inflammation [43,44]. Additionally, our findings showed that transgenic hairy root extract of *Senna obtusifolia* decreased HRV-induced IL-1α expression in both epithelial cells and fibroblasts. The airway epithelium plays a crucial role in the emergence of airway inflammation and remodeling in asthma through its signaling, which draws in and activates immune cells as well as locally present mesenchymal cells. Therefore, the inhibition of IL-1α expression by transformed and transgenic root extracts of *Senna obtusifolia* might modulate inflammatory pathways and, thus, influence further inflammatory agents. It should be noted that betulinic acid, as one of the secondary metabolites present in the tested extracts, also has antiviral properties, which may explain the lack of HRV effect observed in this study [46]. In addition, it may inhibit IL-1 expression [47].

These studies are consistent with ours and support the hypothesis that the tested *Senna obtusifolia* extracts have a positive effect on altering cytokine levels in an in vitro model of rhinovirus-induced cytokines.

The wound healing process is essentially a connective tissue reaction comprising an initial acute inflammatory phase followed by the synthesis of collagen and other intracellular macromolecules, which consequently remodel to form a scar [48]. Both scientific literature and natural folk medicine give many examples of plant species that are used to treat wounds with a positive effect [49]. However, no previous information was found regarding the effect of *Senna obtusifolia* root extract on the wound healing process. Our present phytochemical analysis showed the presence of triterpenoids, anthraquinones, or flavonoids that may have therapeutic potential as anti-inflammatory agents and promoters of wound healing (terpenes or flavonoids), but the one of the compounds in greater quantity was betulinic acid in the tested extract. The transgenic hairy root extract of *Senna obtusifolia* also demonstrated positive effects in the scratch assay, which may be related to the wound healing process. This is consistent with the studies of Nayak et al., who showed that triterpenoid-rich extracts from *Cecropia peltata* and *Pentas lanceolata* were responsible for effective wound healing activity [50,51]. In addition, Kviecinski et al. showed that *Dillenia indica* fruit extract containing betulinic acid (pentacyclic triterpenoid) had a positive effect in psoriasis-like wounds caused by ultraviolet radiation [52]. Similarly, Ebeling et al. noted that birch bark extract (betulin, betulinic acid, lupeol, oleanolic acid, and erythrodiol) accelerates wound healing [53]. This is consistent with our studies, where the pentacyclic triterpene betulinic acid was the main compound in the extract.

Thiols make up the majority of the overall antioxidant defense against reactive oxygen species (ROS) in humans. Together with other antioxidants, total thiols play a critical role in the body’s ability to counteract the effects of ROS on lipid peroxidation. The present study examined the effect of extracts from transformed and transgenic *Senna obtusifolia* roots on total thiol status in cell cultures. The transgenic root extract was found to significantly increase thiol concentration in cells. This may be related to the presence of betulinic acid, which has anti-inflammatory, antiviral, and antitumour properties [40,42,54,55], and is abundant in the transgenic hairy roots of *Senna obtusifolia* [20]. Our present findings suggest it may also have antioxidant and protective properties. The results are consistent with a previous report published by Lingaraju et al. [56], showing that a pretreatment with betulinic acid restored total thiol ranges to normal, thus reversing the depletion of thiols in oxidative lung injury in mice.

Interestingly, incubation with the *Senna obtusifolia* extracts appeared to have no influence on interferon-γ content, although, as expected, HRV-16 caused a significant increase in IFN- γ expression. Previous studies have noted a correlation between measures of asthma severity and IFN-γ production specific to HRV; in addition, both allergens and viruses may result in a defect in Th1 response [57]. It has been suggested that decreased generation of IFN-γ is associated with more severe colds and delayed elimination of viruses. Therefore, finding a compound than can modulate IFN-γ expression could have strong therapeutic implications.

While our study only uses an in vitro model, our results provide valuable information regarding the strong beneficial contribution of biotechnology to the medicinal properties of plants.

## 4. Materials and Methods

### 4.1. Plant Material

Two extracts from transformed (SOA4) and transgenic (SOPSS2 with overexpression of the *PgSS1* gene) *Senna obtusifolia* hairy roots obtained by *Rhozobium rhizogenes*-mediated transformation were used in the study. Hairy roots of *S. obtusifolia* were obtained from axenic plant material grown in vitro from surface sterilized seeds. Using the agrotransformation method, two types of hairy root lines (transformed and transgenic) were inoculated. *Rhizobium rhizogenes* A4 used in the transformation process contained the unmodified (pGFPGUSPlus, a gift from Claudia Vickers (Addgene plasmid # 64401; http://n2t.net/addgene:64401 (accessed on 7 February 2023); RRID:Addgene_64401)) or recombinant (pGFPGUSPlus-PgSS1) plasmid vector. Genetic transformation was carried out on 14-day old *S. obtusifolia* seedlings after cutting the seedlings at the middle of the hypocotyl with a surgical scalpel. The cut site was then inoculated with a properly prepared bacterial suspension, and co-cultivation was carried out in the dark for 3 days. Then, after removing the bacteria, the first hairy roots appeared on the plant material after 10 days. Further selection of transgenic roots and molecular analyzes of the tested clones confirmed that the obtained roots are hairy roots and the material grown further was used to prepare the extracts as described previously [12].

### 4.2. SOA4 and SOPSS2 of Senna obtusifolia Root Extracts Preparation

The extracts were prepared for bioassays as described previously [12]. Briefly, pre-powdered plant materials were extracted in the appropriate solvent and temperature using an ultrasonic bath, and then washed with the same solvent. The extracts were dried, combined, and evaporated under reduced pressure, then lyophilized to dryness and left to dry in the dark for further use.

### 4.3. Cell Cultures

WI-38 were purchased from Sigma-Aldrich (St. Louis, MO, USA) and grown in EMEM medium with 10% fetal bovine serum, 2 mM of L-glutamine, 1% of non-essential amino acids, and standard Penicillin Streptomycin solution (Sigma-Aldrich, St. Louis, MO, USA). The epithelial cell line NHBE was purchased from Lonza (Lonza Walkersville Inc., Walkersville, MD, USA) and cultured in BEGM Bronchial Epithelial Cell Growth Medium BulletKit (Lonza Walkersville Inc., Walkersville, MD, USA). Human Gingival Fibroblasts were obtained from the American Type Culture Collection (ATCC, Manassas, VA, USA); the cells were grown in Dulbecco’s Modified Eagle Medium (DMEM) supplemented with 10% fetal bovine serum, 100 U/mL, penicillin, and 0.1 mg/mL streptomycin. All the experiments (n = 6) were performed after reaching 80–90% confluence (passage three to ten). The viability of the cells was evaluated by Presto Blue (BD Pharmingen, Franklin Lakes, NJ, USA), and the absorbance was measured at 570 nm.

### 4.4. Virus Preparation and Cell Infection

Human Rhinovirus (HRV) 16 was provided by the European Collection of Authenticated Cell Cultures (ECACC, Salisbury, UK). Ohio HeLa cells were infected and a cytopathic effect was observed, with a multiplicity of infection (MOI) of 1, as determined by the literature [58,59]. The target cells were infected by 50 μL of HRV16 or vehicle (medium). The cells were incubated for 24 h (33 °C, 5% CO_2_).

### 4.5. Experimental Procedure

The cultures were exposed to the HRV-16 virus for 24 h (33 °C, 5% CO_2_). Following this, the cells were incubated with *Senna obtusifolia* extracts (0.624 mg/mL) for 24 h (37 °C, 5% CO_2_). The controls were treated with medium only.

### 4.6. RNA Isolation and cDNA Synthesis

Total RNA was isolated using a Total RNA mini kit (A&A Biotechnology, Gdynia, Poland). After purification, the RNA was stored at −80 °C. Following this, 1 μg of RNA was used to perform Reverse transcription using a High Capacity cDNA kit (Applied Biosystems, Foster City, CA, USA). The procedures were performed according to the producers’ protocols.

### 4.7. Gene Expression Analysis

The changes in the expression of *IL-*8*, TNF-α, IL-*1*α*, and *IFN-γ* were assessed using qPCR. TaqMan gene expression assays were used for the selected genes: *IL-*8- Hs00174103_m1, *TNF-α*—Hs00174128_m1, *IL-*1*α*—Hs00174092_m1, *IFN-γ*—Hs01547283_m1 (Life Technologies, Carlsbad, CA, USA). TaqMan analyzer and the 2^−ΔΔCt^ method was used to calculate gene expression and each sample was measured in triplicate. The outcomes were adjusted to an endogenous reference gene (*β-actin*—Hs99999903_m1). The fold change in mRNA expression was calculated by comparing RQ (relative quantification, 2^−ΔΔCt^).

### 4.8. Cytokine Detection in Cells

The protein concentrations (IL-8, TNF-α, IL-1α and IFN-γ) were measured by enzyme-linked immunoabsorbent assay (ELISA) using a commercially-available kit (R&D Systems, Minneapolis, MN, USA) according to the manufacturer’s protocol.

### 4.9. Colorimetric Thiol Detection

The chromogenic reagent for total thiol content determination is 5,5′-dithiobis-(2-nitrobenzoic) acid (DTNB), also known as Ellman’s reagent (Sigma, St. Louis, MO, USA) [60]. In brief, 30 L of each sample was added to 1 mL of PBS and 1 mM of EDTA (pH 7.5), and it was left at room temperature for 30 min. The quantity of 5-thio-2-nitrobenzoic acid (TNB) produced, which was equal to the amount of sulfhydryl groups, was then measured by reading the absorbance at 412 nm. Control samples, which did not include DTNB or protein, were run simultaneously.

### 4.10. In Vitro Wound Healing Potency

The migration of NIH3T3 mouse fibroblasts was assessed using the wound healing scratch assay. According to our lab protocols, NIH3T3 fibroblasts from the 2nd to 6th passage were thawed and cultured in T75 flasks in Dulbecco’s Modified Eagle’s Medium (DMEM) supplemented with 10% Fetal Bovine Serum (FBS) and 1% antibiotics penicillin and streptomycin (P/S) at 37 °C, 95% RH, with a 5% supply of CO_2_ until they reached confluence. Using a Neubauer Hemocytometer, 5 × 10^4^ NIH3T3 cells were seeded separately into 24-well culture plates (in 1ml DMEM medium containing 10% FBS and antibiotics 1% P/S) to a nearly confluent (80%) cell monolayer. Afterwards, they were cultured in starvation medium (DMEM, 1% antibiotics P/S) for 3 h.

The wound was simulated by passing a 200 μL pipette tip through the midline of the confluent cell culture, creating a scratch. After a wash with PBS, various concentrations of SOPSS2 root extract of *Senna obtusifolia* were aliquoted in DMEM media (DMEM, 0.1% BSA, 1% P/S) and passed through a sterile syringe filter (0.22 μm pore size, PES membrane, non-pyrogenic Thermo scientific) prior to addition to the wells. The recovery of the wound was assessed by measuring its width using a micro-camera (soft plus skin analyzer, Callegari, Italy) immediately after the creation of the scratch and at 24 h intervals for 2 days. Attention was paid to measure the exact same frame each time.

The percentage of wound recovery (*WR*%) at any time point (*t*) was calculated using Equation (1):

(1) $$WR\%=\frac{SW_{0}-SW_{t}}{SW_{0}}\times 100$$

where *SW*_0_ and *SW_t_* are the widths of the scratch at time 0 and *t*, respectively

A positive control consisting of 2% FBS was used, together with a negative control consisting of the vehicle (DMEM, 0.1% BSA, 1% P/S). The negative controls showed almost invariant changes in most cases, whereas the positive controls exhibited a slight positive proliferating/migrating ability. All samples and controls were tested twice.

### 4.11. Statistical Analyses

The findings were examined using the Statistica program (v. 8.0, StatSoft, Tulsa, OK, USA). The data distribution was first determined using the Shapiro–Wilk test and the homogeneity of variance using Levene’s test. Following this, the groups were compared using ANOVA with the necessary post hoc tests as a multiple comparison approach. The trends were judged to be significant at *p* < 0.05.

## 5. Conclusions

In conclusion, the extracts tested in our study exhibit anti-inflammatory effects, particularly when derived from transgenic *Senna obtusifolia* hairy root extract, which significantly influence the expression of TNF-α, IL-8, and IL-1α at the gene and protein levels. This study suggests that the tested *Senna obtusifolia* extract might be useful in asthma treatment, although additional research is required. It may also represent a potential therapeutic agent for skin wound healing, supporting its use in traditional medicine. Additionally, our research also shows that application of biotechnology strategies can be useful in enhancing the natural properties of medicinal plants.

## Data Availability

The data presented in this study are available on request from the corresponding author. The data are not publicly available due to restrictions eg privacy or ethical.
